# Supplementary material for: N123I mutation in the ALV-J receptor-binding domain region enhances viral replication ability by increasing the binding affinity with chNHE1
Source: PLoS Pathog. 2024 Feb 7;20(2):e1011928. doi: 10.1371/journal.ppat.1011928 (PMC10878525; doi:10.1371/journal.ppat.1011928)
Supplement: S1 Table — (PDF) [file ppat.1011928.s001.pdf]

S1 Table. The ALV-J strains were used in the study.

Strains	Origin	Host	Accession no	Strains	Origin	Host	Accession no
BR119	Shandong	layer	KF562373	UD4	USA	broiler	AF307951
HB201101	Hebei	layer	MW476816	UD5	USA	broiler	AF307952
CAUHM01	Beijing	layer	HM640944	YZ9902	Jiangsu	broiler	HM235670
CAUSY01	Beijing	layer	HM640944	GD06SL1	Guangdong	local	EF107624
CAUXT01	Beijing	layer	HM640947	GD06SL2	Guangdong	local	EF103129
CAUYL01	Beijing	layer	HM640948	GD06SL3	Guangdong	local	EF103130
GD1109	Guangdong	layer	JX254901	GD06SL4	Guangdong	local	EF103131
GDHN-YH1	Guangdong	layer	MK683480	GD1406-H	Guangdong	local	KU500033
HAY013	Jiangsu	layer	HM235665	GD1407-L	Guangdong	local	KU500034
HB201102-1	Hebei	layer	MW476819	GD1408-2	Guangdong	local	KU500037
HB2020	Hubei	layer	ON840093	GD14J2	Guangdong	local	KU500032
HLJ09MDJ-1	Heilongjiang	layer	JN624880	GD15MM01	Guangxi	local	MN066152
HLJ09SH01	Heilongjiang	layer	HQ634806	GD19GZ02	Guangdong	local	MN893851
HLJ09SH02	Heilongjiang	layer	HQ634807	GDQY1201	Guangdong	local	JX423792
HLJ13SH01	Heilongjiang	layer	KM376510	GDYH-B1	Guangdong	local	MK940585
HN1001-3	Henan	layer	HQ260976	GX14HG01	Guangxi	local	KU997685
HUB09WH03	Hubei	layer	HQ634805	GX14HG04	Guangxi	local	KX058878
JL08CH3-1	Jilin	layer	HQ634809	GX14LT07	Guangxi	local	KX034517
JL093-1	Jilin	layer	JN624878	GX14NN01	Guangxi	local	MN066154
JL10HW01	Jilin	layer	HQ634800	GX14YL03	Guangxi	local	KR025484
JL10HW02	Jilin	layer	HQ634801	GX15JL01	Guangxi	local	MN735294
JS09GY2	Jiangsu	layer	GU982307	GX15MM61	Guangxi	local	MN066150
JS09GY3	Jiangsu	layer	GU982308	GX15MM6-2	Guangxi	local	KU934276
JS09GY5	Jiangsu	layer	GU982309	GX16MM92	Guangxi	local	MN066149
JS09GY6	Jiangsu	layer	GU982310	GX16NN04	Guangxi	local	MN735296
LN08SY10	Liaoning	layer	HQ634802	GX16YL01	Guangxi	local	MN735297
LN09SY31	Liaoning	layer	HQ634803	GX16YL02	Guangxi	local	MN066147
SCDY1	Sichuan	layer	HQ425636	GX16ZS01	Guangxi	local	KY983549
SD09DP03	Shandong	layer	JN624879	GX17NN05	Guangxi	local	MN066145
SD09DP04	Shandong	layer	HQ634808	GX17YL01	Guangxi	local	MN066143
SD100501J	Shandong	layer	HQ270189	GX17YL101J	Guangxi	local	MZ393159
SD100502J	Shandong	layer	HQ270188	GX18NN02	Guangxi	local	MN066140
SD1009	Shandong	layer	KF768000	GX19NN05J	Guangxi	local	MZ393174
sdau1001	Shandong	layer	JN389517	GX19YL09J	Guangxi	local	MZ393169
sdau1002	Shandong	layer	JN389518	GX20YL05	Guangxi	local	MW491266
SDAU1005	Shandong	layer	KF562375	GX20YL09	Guangxi	local	MW491270
SDAU1102	Shandong	layer	KU159178	GX20YL12J	Guangxi	local	MZ393181
SX090912J	Shanxi	layer	HQ386988	GX20YL16J	Guangxi	local	MZ393191
SX090915J	Shanxi	layer	HQ386989	GX20YL40J	Guangxi	local	MZ393185
HB09-1	Hebei	layer	HM600812	GX20YL42J	Guangxi	local	MZ393184
4817	USA	broiler	AF247385	GX-J-4	Guangxi	local	JQ246093
6683	USA	broiler	AF247387	GX-J-6	Guangxi	local	JQ246095

6803	USA	broiler	AF247389	GX-J-7	Guangxi	local	JQ246096
10075-16	USA	broiler	GU222406	GX-J-8	Guangxi	local	JQ246097
10075-20	USA	broiler	GU222407	GX-J-10	Guangxi	local	JQ246099
ADOL-7501	USA	broiler	AY027920	GX-RJ01	Guangxi	local	MT119963
AF88	USA	broiler	AF247390	HB18XH01	Hubei	local	MN735298
BJ0301	Beijing	broiler	AY897230	HN17ZZ01	Hunan	local	MN735299
CAUGX01	Beijing	broiler	JF931999	HN17ZZ02	Hunan	local	MN735300
GD13GZ	Guangdong	broiler	KU500030	JS13NT01	Jiangsu	local	MN735301
GD1411-1	Guangdong	broiler	KU500038	JS14NT02	Jiangsu	local	MN735302
GD1411-2	Guangdong	broiler	KU500039	JS16JH3	Jiangsu	local	MG700535
GD1411-4	Guangdong	broiler	KU500041	JS16JH5	Jiangsu	local	MG700537
GD16HZ02	Guangdong	broiler	MT538252	JS18YZ01	Jiangsu	local	MN735303
GD16JM01	Guangdong	broiler	MT538251	LC110515-3	Shandong	local	GU982308
GD17HZ01	Guangdong	broiler	MT538238	LC110515-4	Shandong	local	KC841153
GD18JM01	Guangdong	broiler	MT538241	M180	Guangdong	local	KX611834
GD18SG01	Guangdong	broiler	MT538242	SH18JY01	Shanghai	local	MN735306
GD18ZH01	Guangdong	broiler	MT538243	SH18JY02	Shanghai	local	MN735307
GD19ZH02	Guangdong	broiler	MT538249	WN100401	Anhui	local	HQ271447
GD19FS01	Guangdong	broiler	MT538245	WN100402	Anhui	local	HQ271448
HB1801	Hebei	broiler	MN419352	GD17FS01	Guangdong		MN262599
Hc1	USA	broiler	AF247391	GD17FS03	Guangdong		MN262601
HPRS103	UK	broiler	Z46390	GD18HZ12	Guangdong		MN262610
JL1801	Jilin	broiler	MN419348	GD18HZ14	Guangdong		MN262612
JL1802	Jilin	broiler	MN419349	GD18QY20	Guangdong		MN262618
JL1803	Jilin	broiler	MN419350	GD19JY28	Guangdong		MN262626
JL1804	Jilin	broiler	MN419351	HN1001-1	Beijing		HQ260974
JS1805	Jiangsu	broiler	MN419339	HN1001-2	Beijing		HQ260975
JS1806	Jiangsu	broiler	MN419340	HuB09-1	Hubei		HM600813
JS1808	Jiangsu	broiler	MN419342	HUB09WH02	Hubei		HQ634804
JS-nt	Jiangsu	broiler	HM235667	JGD18QY19	Jiangxi		MN262617
LH20180301	Shandong	broiler	MK944404	JGD18QY22	Jiangxi		MN262620
LN1801	Liaoning	broiler	MN419354	JS14XJ04	Jiangsu		MT783255
NX0101	Ningxia	broiler	AY897227	JS15LYJ01	Jiangsu		MT783269
PDRC-59831	USA	broiler	KP284572	JS16DJ01	Jiangsu		MT783276
SD0001	Shandong	broiler	AY897223	JS16JH2	Jiangsu		MG700534
SD0002	Shandong	broiler	AY897224	JX19AY10	Jiangxi		MN262549
SD1802	Shandong	broiler	MN419336	JX19AY11	Jiangxi		MN262550
SD1803	Shandong	broiler	MN419337	JX19AY17	Jiangxi		MN262556
SD1804	Shangdong	broiler	MN419338	JX19AY27	Jiangxi		MN262566
SD1809	Shandong	broiler	MN419346	JX19DX02	Jiangxi		MN262570
SD1810	Shandong	broiler	MN419344	JX19DX19	Jiangxi		MN262587
SD9902	Shandong	broiler	AY897221	JX19GF05	Jiangxi		MN262587
SDGM1801	Shandong	broiler	MN413674	JX19TH06	Jiangxi		MN262533
TJ1801	Tianjing	broiler	MN419353	JX19TH07	Jiangxi		MN262534

UD2	USA	broiler	AF307949	JX19TH09	Jiangxi	MN262536

Abbreviation: ALV-J, Avian leukosis virus subgroup J.

[1] ALV-J isolate hosts: broiler, broiler chicken, layer: layer chicken, local: local chicken
