# Supplementary material for: N123I mutation in the ALV-J receptor-binding domain region enhances viral replication ability by increasing the binding affinity with chNHE1
Source: PLoS Pathog. 2024 Feb 7;20(2):e1011928. doi: 10.1371/journal.ppat.1011928 (PMC10878525; doi:10.1371/journal.ppat.1011928)
Supplement: Attachment — Submitted filename: response_to_reviewer_comments.docx [file ppat.1011928.s002.docx]

**Point-by-point responses to the reviewers' comments:**

**Response to reviewer #1:**

In the present manuscript “N123I mutation in the ALV-J receptor-binding domain region enhances the viral replication ability by increasing binding affinity with chNHE1”, Yu et al. analyzed the effect of a single amino-acid substitution in the ALV-J gp85 on in vitro virus replication and receptor binding. The authors compared the prototype ALV-J isolate HPRS103 and the recent Chinese isolate JL08CH3-1 and mapped the difference to GP85. Subsequently, they reproduced the difference by substituting N123I within the HPRS103 envelope. Yu et al. attributed this to the rapid ALV-J radiation in China and compare it with SARS-CoV-2 evolution in recent years. Overall, the main finding of the study is correct, but should be better supported by experiments and its relevance for the virus evolution should be clearly explained. The N123I is prevalently present in recent Chinese isolates from laying chicken breeds, but also in isolates from broilers, both in British isolates closely related to HPRS103 and in highly pathogenic American isolates. Thus, the N123I does not explain either the host range extension of the virus from broilers to egg-type chickens or the recent gp85 variation in Asia. Much of the background information and discussion is, therefore, irrelevant and should be deleted or replaced by a more plausible context. I also suggest (in accordance to the aforementioned conceptual flaw) that Fig. 7 is put into Results and commented in a new chapter.

**Responses:** We thank the reviewer for a careful review of our manuscript and for the insightful suggestions. We deleted or modified some irrelevant background information in the introduction and discussion in the revised manuscript. For example, we revised several sentences in lines 49, 57, 58, 59, 61, 70, and 82 in the introduction and in lines 290 and 303-305 in the discussion. We also replaced the sentence 'Its host range has expanded from broilers to layer chickens and local chickens, seriously affecting the safety of poultry breeding sources in China' with 'ALV-J is the dominant ALV subgroup in chickens, which seriously affects the safety of poultry breeding sources in China' in lines 295–296.

The purpose of Figure 7 in the manuscript is to analyze the potential correlation between the N123I mutation in ALV-J-gp85 and the outbreak of ALV-J, providing guidance for future epidemiological investigations of ALV-J. Compared with Figures 1 to 6, there is no substantive experimental research content in Figure 7. We only collected and downloaded a large number of gp85 sequences of ALV-J strains for phylogenetic and amino acid sequence analysis. Considering the consistency and coordination of the article, we still placed Figure 7 in the discussion section. However, based on your suggestion, we have provided detailed information of ALV-J strains used in Figure 7 for constructing phylogenetic trees in the supplementary materials.

**Major Issues:**

1. The virus replication in vitro was tested by virus titration, but the infection was performed at an extremely low dose of the virus (MOI 0.01). Under such conditions, the experiment can be easily biased by inaccurately measured titer of the virus stock (two virus variants with potentially different replication capacity). Virus replication should be quantified in parallel using an RT assay.

**Responses**: Thank you very much for your valuable suggestions. ALV-J, as a retrovirus, has a relatively low viral titer compared to ALV-A (1) and other avian viruses (IBDV, NDV, FDV4, and AIV). In viral replication experiments, most studies choose an inoculation dose of 0.003-0.01 MOI (2-3). Therefore, we selected a dose of 0.01 MOI for virus replication experiments in this study.

According to your suggestion, to increase the reliability of the results, we detected the RT activity of JL08CH3-1/HPRS103, rHPRS/JL3-1/rHPRS103, and rHPRS103-N123I/rHPRS103 at 1–7 dpi using a colorimetric reverse transcriptase assay. The results showed that at 3–7 dpi, the RT activity of JL08CH3-1 was higher than that of the prototype strain HPRS103 (Fig 1B) and the RT activity of rHPRS/JL3-1was higher than that of rHPRS103 (Fig 1F), similar to the results of viral titers. We have added the results in the section 'Replication advantage of JL08CH3-1 strain is associated with gp85' (Lines 98–101 and 117–120) and Fig 1B and 1F of Figure 1. In addition, the results showed that the RT activity of rHPRS103-N123I was higher than that of rHPRS103 at 3–7 dpi (Fig 6E), consistent with the viral titers results, further confirming that the N123I mutation in gp85 increases the replication ability of ALV-J. We also added the results (lines 213–214) and Fig 6E to the revised manuscript.

**Fig 1B**

**Fig 6E**

**Fig 1F**

**Figure legends**

Fig 1B, RT assay. DF1 cells are infected with the layer strain JL08CH3-1 or HPRS103 at an MOI of 0.01, which are harvested and quantified using a colorimetric reverse transcriptase assay at 1, 2, 3, 4, 5, 6, and 7 dpi. Fig 1F, RT assay. DF1 cells are infected with the 0.01 MOI rHPRS103-N123I or rHPRS103, which are harvested and quantified using a colorimetric reverse transcriptase assay at 1, 2, 3, 4, 5, 6, and 7 dpi. Fig 6E. The replication ability of rHPRS103-N123I and rHPRS103 is detected using RT assay. DF1 cells are incubated with rHPRS103-N123I and rHPRS103 at an MOI of 0.01, and then harvested and quantified at 1, 2, 3, 4, 5, 6, and 7 dpi.

**References**

1) Maas R, van Zoelen D, Oei H, Claassen I. Replacement of primary chicken embryonic fibroblasts (CEF) by the DF-1 cell line for detection of avian leucosis viruses. Biologicals. 2006; 34(3):177–81. https://doi.org10.1016/j.biologicals.2005.09.002 PMID: 16257542.
2) Li T, Xie J, Yao X, Zhang J, Li C, Ren D, et al. The tyrosine phosphatase SHP-2 dephosphorylated by ALV-J via its Env efficiently promotes ALV-J replication. Virulence. 2021; 12(1):1721–1731. <https://doi.org10.1080/21505594.2021.1939952> PMID: 34167452.
3) Qu Y, Liu L, Niu Y, Qu Y, Li N, Sun W, et al. Viral proliferation and expression of the tumor-related gene in different chicken embryo fibroblasts infected with different tumorigenic phenotypes of avian leukosis virus subgroup J. Poult Sci. 2016; 95(10):2383–90. <https://doi.org>/10.3382/ps/pew180 PMID: 27609806.
2. The binding capacity of JL08CH3-1 gp85 to chNHE1 was tested using protein-cell binding assay. However, virus-cell binding assay would be more direct and biologically relevant.

**Responses:** We thank the reviewer for insightful suggestions. The protein-cell binding assay is a common technique for measuring protein-binding affinities by using flow cytometry to analyze ligand binding to proteins present on the surface of a cell (1). The assay has been widely used for the binding of SARS-coronavirus S1 protein with its receptor ACE2 (2-4). In addition, the assays have also been used in our previous studies (5-8) and other studies on the binding of the gp85 of ALV with its receptor (9). For example, we used this assay to detect the binding between the gp85 of ALV-J and its receptor chNHE1, as well as the binding between the gp85 of ALV-A and ALV-K and its receptor Tva (5-8). Therefore, in this study, we also used the protein-cell binding assays to detect the binding capacity of the gp85 of ALV-J to chNHE1.

**References**

1) Hunter SA, Cochran JR. Cell-Binding assays for determining the affinity of protein-protein interactions: technologies and considerations. Methods Enzymol. 2016; 580:21–44. https://doi.org/10.1016/bs.mie.2016.05.002. PMID: 27586327.
2) Li W, Zhang C, Sui J, Kuhn JH, Moore MJ, Luo S, et al. Receptor and viral determinants of SARS-coronavirus adaptation to human ACE2. EMBO J. 2005; 24(8):1634–43. https://doi.org/10.1038/sj.emboj.7600640 PMID: 15791205.
3) Tian F, Tong B, Sun L, Shi S, Zheng B, Wang Z, et al. N501Y mutation of spike protein in SARS-CoV-2 strengthens its binding to receptor ACE2. Elife. 2021; 10:e69091. <https://doi>.org/ 10.7554/eLife.69091 PMID: 34414884.
4) He Y, Li J, Jiang S. A single amino acid substitution (R441A) in the receptor-binding domain of SARS coronavirus spike protein disrupts the antigenic structure and binding activity. Biochem Biophys Res Commun. 2006; 344(1):106–13. [https://doi.org/10. 1016/](https://doi.org/10.%201016/)j.bbrc.2006.03.139 PMID: 16615996.
5) Guan X, Zhang Y, Yu M, Ren C, Gao Y, Yun B, et al. Residues 28 to 39 of the extracellular loop 1 of chicken Na^+^/H^+^ exchanger type I mediate cell binding and entry of subgroup J avian leukosis virus. J Virol. 2017; 92(1):e01627-17. https://doi.org/10.1128/JVI.01627–17 PMID: 29070685.
6) Zhang Y, Yu M, Xing L, Liu P, Chen Y, Chang F, et al. The bipartite sequence motif in the N and C termini of gp85 of subgroup J avian leukosis virus plays a crucial role in receptor binding and viral entry. J Virol. 2020; 94(22):e01232–20. <https://doi.org/10.1128/JVI>. 0123 2-20 PMID: 32878894.
7) Chen Y, Wang S, Li X, Yu M, Liu P, Meng L, et al. Residues L55 and W69 of Tva mediate entry of subgroup A avian leukosis virus. J Virol. 2022; 96(18):e0067822. https://doi. org/10.1128/jvi.00678-22 PMID: 36069550.
8) Li X, Chen Y, Yu M, Wang S, Liu P, Meng L, et al. Residues E53, L55, H59, and G70 of the cellular receptor protein Tva mediate cell binding and entry of the novel subgroup K avian leukosis virus. J Biol Chem. 2023; 299(3):102962. https://doi.org/ 10.1016/j.jbc.2023.102962. PMID: 3671707.
9) Li J, Chen J, Dong X, Liang C, Guo Y, Chen X, et al. Residues 140-142, 199-200, 222-223, and 262 in the surface glycoprotein of subgroup A avian leukosis virus are the key sites determining Tva receptor binding affinity and infectivity. Front Microbiol. 2022; 13:868377. <https://doi.org/10.3389/fmicb.2022.868377> PMID: 35572683.

**Minor Issues**

Reviewer #1: Minor points

1. Abstract, line 19; Introduction, line 53. ALV-J is not present worldwide. Europe and the United States are ALV-J-free now.

**Responses**: We thank the reviewer for their careful view. Based on your advice and extensive literature review, we have deleted the relevant sentences in the revised manuscript (line 51).

2. Abstract, line 26, 27. It should be consistently said that structural modeling as performed in this study is merely indicative and the results should be evaluated carefully.

**Responses:** We thank the reviewer for a careful review of our manuscript and for insightful suggestions. The open-source server trRosetta (https://yanglab.nankai.edu.cn/trRosetta/), used in this study, is relatively accurate in predicting protein structure (1). However, as you mentioned, because there is currently no homologous model for the gp85 protein of ALV, the structural modeling in this study is predicted. Therefore, based on your suggestion, we replaced 'revealed' with 'indicated' in the abstract (line 26) section, replaced 'revealed' with 'suggested' (line 198) and 'significantly increased' with 'was closely related to the improved' (lines 198 and 199) in the result section and added 'might' in the discussion (lines 278 and 291) to improve the accuracy of the results.

**References**

1) Du Z, Su H, Wang W, Ye L, Wei H, Peng Z, et al. The trRosetta server for fast and accurate protein structure prediction. Nat Protoc. 2021; 16(12):5634–5651. <https://doi>. org/10 .1038/ s41596-021-00628-9 PMID: 34759384.
3. Introduction is rather long and trivial textbook facts (such as lines 70-72) could be deleted. Reference 23 does not document the statement before.

**Responses:** Thank you very much for your suggestion. We have deleted the sentence in the revised manuscript. In addition, we have revised several sentences in lines 49, 57, 58, 59, 61, 70, and 82 in the introduction. At the same time, we revised Reference 23 to the right Reference.

**Reference**

23) Poehlmann S, Simmons G. Viral entry into host cells preface[J]. Adv Exp Med Biol. 2013; 790:V-VI. https://doi.org/10.1007/978-1-4614-7651-1.

4. In the chapter Binding capacity of JL08CH3-1...., the terms binding capacity, binding capability, binding rate, and binding ability are confused (same also in Fig. 2). Overall, the chapter is poorly written with a lot of unnecessary detail.

**Responses:** We thank the reviewer for a careful review of our manuscript. According to the suggestions, we replaced the 'binding capability' and 'binding ability' with 'binding capacity' in the revised manuscript (lines 253 264, 266, 267, 270, 275, 292, and 317). In addition, in the chapter 'Binding capacity of JL08CH3-1-gp85 to chNHE1 surpasses that of HPRS103-gp85 to chNHE1', we evaluated the binding capacity of gp85 proteins to chNHE1-expressing 293T cells with FACS, and we used binding rate to demonstrate the binding capacity of gp85 to chNHE1. Therefore, the binding rate was used in this section and Fig 2A (lines 128, 129, and 131).

5. Results, line 158. The reference 36 seems to be incorrect at this place.

**Responses:** We thank the reviewer for pointing this out. We revised reference 36 at this place in the revised manuscript.

**Reference**

36) Zhang Y, Yu M, Xing L, Liu P, Chen Y, Chang F, et al. The bipartite sequence motif in the N and C termini of gp85 of subgroup J avian leukosis virus plays a crucial role in receptor binding and viral entry. J Virol. 2020; 94(22):e01232–20. https://doi.org/10.1128/JVI.01232-20 PMID: 32878894.

6. Discussion, lines 310-313. These two sentences do not make good sense.

**Responses:** We thank the reviewer for a careful view of our manuscript. We have made modifications to these two sentences in the revised manuscript (lines 303–305).

7. Methods, line 390. The concentration of the cell extract with gp85 should be given.

**Responses:** We thank the reviewer for the suggestion. The protein concentration (200 ng/μL) has been added in lines 383 and 384.

8. Fig. 3B. Description of gp85 mutants does not correspond to the text and the alignment 3A.

**Responses:** We thank the reviewer for a careful view of our manuscript. We have rechecked and supplemented the bands of E239K, R271S, and Q282R mutant proteins in Fig 3B.

Fig 3B

**Response to reviewer #2:**

In this study, Yu and colleagues have found a strong linkage between the heightened replication efficacy of the layer strain JL08CH3-1 and the enhanced affinity exhibited by gp85 towards receptor chNHE1 compared to the prototype strain HPRS103. Particularly, the N123I mutation within the RBD region of gp85 precipitates an elevation in the binding affinity between gp85 and the receptor. They further demonstrated that N123I increased the binding capacity between gp85 and chNHE1, likely through enhancing the stability of gp85, expanding the interaction interface, and increasing the number of hydrogen bonds at the interaction interface. Furthermore, they systematically analyzed the amino acid sequence of ALV-J and conducted animal experiments, revealing that the N123I mutation enhanced the replication ability of ALV-J, making it more easily transmitted and likely to cause large epidemics. In general, these systematic studies are well-designed and the results make a valuable contribution to our understanding of the importance of amino acid mutations within the RBD region of the ALV-J gp85 protein in the viral evolution process. However, the manuscript should be improved before its acceptance for publication.

**Major Issues:**

1. Why did you choose the layer strain JL08CH3-1 as the representative strain for this study?

**Responses:** We thank the reviewer for a careful view of our manuscript. Since 2008, ALV-J has been widely prevalent the layer chickens aged 15–22 weeks in China, inducing different types of tumors in the liver, kidneys, and spleen (1-2). Phylogenetic analysis showed that the env gene of the ALV-J layer strains formed a relatively independent group, with less than 90.6% homology with the prototype strain HPRS103 (1). JL08CH3-1 is one of the ALV-J layer strains isolated from layer chickens with lymphocytic leukemia and hemangioma (1). The env gene of JL08CH3-1 has a high homology with the env gene of most ALV-J layer strains. Therefore, in this study, the layer strain JL08CH3-1 was selected as a representative of ALV-J layer strains.

**References**

1) Pan W, Gao Y, Qin L, Ni W, Liu Z, Yun B, et al. Genetic diversity and phylogenetic analysis of glycoprotein GP85 of ALV-J isolates from Mainland China between 1999 and 2010: coexistence of two extremely different subgroups in layers. Vet Microbiol. 2012;156(1-2):205-12. https://doi.org 10.1016/j.vetmic.2011.10.019 PMID: 22101092.
2) Gao Y, Yun B, Qin L, Pan W, Qu Y, Liu Z, et al. Molecular epidemiology of avian leukosis virus subgroup J in layer flocks in China. J Clin Microbiol. 2012; 50(3):953–60. https://doi.org/10.1128/JCM.06179-11 PMID: 22205787.
2. Why do you directly analyze the impact of gp85 on replication when you find that the replication ability of the broiler strain is stronger than that of the prototype strain?

**Responses:** We thank the reviewer for a careful view of our manuscript. As described in the introduction, gp85 is the surface protein of the ALV, which directly binds to the receptor and mediates ALV entry into the host cells (1-2). Our previous results showed that compared with the prototype strain HPRS103, there was significant variations in the RBD region of gp85 protein of ALV-J layer strains (3-4). Meanwhile, ALV-J layer strains showed higher replication ability in cells than that of HPRS103 (5). Many studies have demonstrated that amino acid mutations in the RBD region of surface glycoprotein may directly enhance the binding capacity of env proteins to receptors to promote viral replication (6-9). Therefore, based on these research findings, we speculate that the enhanced replication ability of the ALV-J layer strain is related to the genetic variation of the gp85 gene.

**References**

1) Chai N, Bates P. Na^+^/H^+^ exchanger type 1 is a receptor for pathogenic subgroup J avian leukosis virus. Proc Natl Acad Sci U S A. 2006; 103(14):5531–6. https://doi.org/ 10.1073 /pnas. 0509785103 PMID: 16567631.
2) Federspiel MJ. Reverse engineering provides insights on the evolution of subgroups A to E avian sarcoma and leukosis virus receptor specificity. Viruses. 2019;11(6):497. https://doi.org10.3390/v11060497 PMID: 31151254.
3) Gao Y, Yun B, Qin L, Pan W, Qu Y, Liu Z, et al. Molecular epidemiology of avian leukosis virus subgroup J in layer flocks in China. J Clin Microbiol. 2012; 50(3):953–60. https://doi.org/10.1128/JCM.06179-11 PMID: 22205787.
4) Pan W, Gao Y, Qin L, Ni W, Liu Z, Yun B, et al. Genetic diversity and phylogenetic analysis of glycoprotein GP85 of ALV-J isolates from Mainland China between 1999 and 2010: coexistence of two extremely different subgroups in layers. Vet Microbiol. 2012;156(1-2):205–12. https://doi.org 10.1016/j.vetmic.2011.10.019 PMID: 22101092.
5) Qu Y, Liu L, Niu Y, Qu Y, Li N, Sun W, et al. Viral proliferation and expression of tumor-related gene in different chicken embryo fibroblasts infected with different tumorigenic phenotypes of avian leukosis virus subgroup J. Poult Sci. 2016; 95(10):2383–90. https://doi.org/10.3382/ps/pew180 PMID: 27609806.
6) Hou YJ, Chiba S, Halfmann P, Ehre C, Kuroda M, Dinnon KH, et al. SARS-CoV-2 D614G variant exhibits efficient replication ex vivo and transmission in vivo. Science. 2020; 370(6523):1464–1468. https://doi.org/10.1126/science.abe8499 PMID: 33184 236.
7) Ozono S, Zhang Y, Ode H, Sano K, Tan TS, Imai K, et al. SARS-CoV-2 D614G spike mutation increases entry efficiency with enhanced ACE2-binding affinity. Nat Commun. 2021; 12(1):848. https://doi.org/10.1038/s41467-021-21118-2 PMID: 33558493.
8) Li H, Wang S, Kong R, Ding W, Lee FH, Parker Z, et al. Envelope residue 375 substitutions in simian-human immunodeficiency viruses enhance CD4 binding and replication in rhesus macaques. Proc Natl Acad Sci U S A. 2016; 113(24):E3413–22. <https://doi.org/10>. 1073/pnas.1606636113 PMID: 27247400.
9) Hueffer K, Govindasamy L, Agbandje-McKenna M, Parrish CR. Combinations of two capsid regions controlling canine host range determine canine transferrin receptor binding by canine and feline parvoviruses. J Virol. 2003; 77(18):10099–105. <https://doi.org/10>. 1128/jvi.77.18.10099-10105.2003 PMID: 12941920.

**Minor Issues**

1 Line 62, replace 'show' with ' shows'.

**Responses:** We thank the reviewer for a careful view of our manuscript. We have replaced the 'show' with 'shows' in the revised manuscript (line 58).

2 2. Line 85, It is necessary to add abbreviations for simian-human immunodeficiency virus and feline panleukopenia virus, such as severe acute respiratory syndrome coronavirus 2 (SARS-CoV-2)

**Responses:** We thank the reviewer for this suggestion. We have added the abbreviations of simian-human immunodeficiency virus (SHIV) and feline panleukopenia virus (FPV) in line 78.

3 Line 105 and 106, replace ' log10 ' with 'log_10_ '.

**Responses:** We thank the reviewer for a careful view of our manuscript. We have replaced ' log10 ' with 'log_10_ ' in lines 96, 97,116 and 117.

4 How do you get the binding free energies of HPRS103-gp85complexed with TM-chECL1(-300 kJ/mol) or N123I-gp85 complexed with TM-chECL1(-600 kJ/mol), which is an average or a numerical value at a certain point?

**Responses:** We thank the reviewer for a careful view of our manuscript. The binding free energies of HPRS103-gp85 complexed with TM-chECL1(-300 kJ/mol) or N123I-gp85 complexed with TM-chECL1(-600 kJ/mol) was an average value after their structures reached equilibrium.

5 Line 225, replace 'enhanced' with 'enhances'.

**Responses:** We apologize for this typographical error. We have replaced 'enhanced' with 'enhances' in line 224.

6 Line 241, replace 'ALV-gp85' with 'ALV-J gp85'.

**Responses:** We thank the reviewer for pointing this out. We have replaced 'ALV-gp85' with ' ALV-J gp85' in line 239.

7 Line 413, the Reed and Muench method, reference should be cited.

**Responses:** We thank the reviewer for a careful view of our manuscript. We have added reference 67 for the Reed and Muench method (line 422).

**Reference:**

67) Reed L J, Muench H. A simple method of estimating fifty percent endpoints. Am J Hyg. 1938; 27(3):493–49. https://doi.org/10.1093/oxfordjournals.aje.a118408.

8 Structural analysis in material methods requires reference to support the credibility of the prediction method.

**Responses:** We thank the reviewer for this suggestion. We have added reference 69 (line 450), reference 70 (line 453), and reference 71 (line 456) to support the credibility of the prediction method.

**Reference:**

69) Du Z, Su H, Wang W, Ye L, Wei H, Peng Z, et al. The trRosetta server for fast and accurate protein structure prediction. Nat Protoc. 2021; 16(12):5634–5651. <https://doi>. org/10.1038/ s41596-021-00628-9 PMID: 34759384.

70) Yan Y, Zhang D, Zhou P, Li B, Huang SY. HDOCK: a web server for protein-protein and protein-DNA/RNA docking based on a hybrid strategy. Nucleic Acids Res. 2017; 45(W1): W365–W373. https://doi.org/10.1093/nar/gkx407PMID: 28521030.

71) Laskowski RA, Swindells MB. LigPlot+: multiple ligand-protein interaction diagrams for drug discovery. J Chem Inf Model. 2011; 51(10):2778–86. https://doi.org/10.1021/ci200227u PMID: 21919503.

9 The labeling of the catalog numbers, companies, and origins of reagents and instruments within the Materials and Methods section is inconsistent.

**Responses:** We are sorry for this omission. We have standardized the labeling of reagents and instruments in the Materials and Methods section (lines 386, 387, 390–392, 398, 402, 407, 419, 427, 442, 446, and 447).

10 Fig 1A,1D, and 6D replacing ' LgTCID50/ml' with ' Log10TCID50/ml ' to be consistent with the description in the article. The '（）' of Fig1A should not be bolded.

**Responses:** We thank the reviewer for a careful view of our manuscript. We have made modifications to the Fig 1A, 1D, and 6D based on your suggestions.

11 Fig 1C and 6C legend, Scale bar needs to be added.

**Responses:** We thank the reviewer for a careful view of our manuscript. Because we added the RT activity results of JL08CH3-1/HPRS103 in Fig 1B, the IFA of rHPRS/JL3-1 and rHPRS103 was re-labeled as Fig 1D. So, we have added the Scale bar at the legend of Fig 1D (line 755) and 6C (line 825).

12 The format of the reference is not uniform, such as the format of author name (ref 1 VS ref 1), which needs to be modified according to the Author guide.

**Responses:** We thank the reviewer for pointing this out. We have standardized the format of the reference.

13 The manuscript requires extensive revision for grammar and style issues.

**Responses:** We thank the reviewer for their careful view. This manuscript was proofread by an English-speaking professional with a scientific background at Editage (www.editage.cn) for English language editing.
