# Supplementary material for: N123I mutation in the ALV-J receptor-binding domain region enhances viral replication ability by increasing the binding affinity with chNHE1
Source: PLoS Pathog. 2024 Feb 7;20(2):e1011928. doi: 10.1371/journal.ppat.1011928 (PMC10878525; doi:10.1371/journal.ppat.1011928)
Supplement: Attachment — Submitted filename: response_to_reviewer_comments.docx [file ppat.1011928.s003.docx]

**Point-by-point responses to the reviewers' comments:**

**Response to reviewer #1:**

In the present manuscript “N123I mutation in the ALV-J receptor-binding domain region enhances the viral replication ability by increasing binding affinity with chNHE1“, Yu et al. analyzed the effect of a single amino-acid substitution in the ALV-J gp85 on in vitro virus replication and receptor binding. The authors compared the prototype ALV-J isolate HPRS103 and the recent Chinese isolate JL08CH3-1 and mapped the difference to GP85. Subsequently, they reproduced the difference by substituting N123I within the HPRS103 envelope. In the revised version, the authors have substantially improved the manuscript PPATHOGENS-D-23-01396R1. However, some of my criticisms and reservations have not been fully addressed and I suggest another revision of the manuscript. Overall, the main finding of the study is correct, but some partial conclusions should be better supported by experiments with a more direct biological relevance.

**Major Issues:**

1. I recommend that the binding capacity of JL08CH3-1 gp85 to chNHE1 should be tested using virus-cell binding assay in addition to the protein-cell binding assay. This experiment is not demanding and adds the biological relevance to the results. The protein-cell binding assay does not apply the gp85 in trimeric form and the results of both assays usually differ.

**Responses**: We appreciate the valuable suggestions provided by the reviewers. In line with your recommendations, we conducted virus-cell binding assays using RT-qPCR and FCAS assays to improve the reliability of our results that the binding capacity of JL08CH3-1 gp85 or N123I-gp85 to chNHE1 was stronger than that of HPRS103-gp85. Results from the virus-cell binding assays using qRT-PCR and FACS showed that rHPRS/JL3-1 bound to chNHE1 7.96-fold and 1.29-fold more effectively than rHPRS103 (Fig 2B, C). We have included new findings in the section titled "The binding capacity of JL08CH3-1-gp85 to chNHE1 exceeds that of HPRS103-gp85 to chNHE1"(lines 132-135) and 2B and 2C of Figure 2. Furthermore, the results from our virus-cell binding assays using qRT-PCR and FACS indicate that rHPRS103-N123I has a binding capacity to chNHE1 that is 7.14 and 1.27 times higher than that of rHPRS103, as shown in Fig 6D and 6E. We have also included these results (lines 212-216) and Fig 6D and 6E in the revised manuscript.

**Minor Issues**

Reviewer #1: Minor points

1. In the introduction, the sentence ''Epidemiological investigations have shown... '' (lines 57-58) does not make good sense.

**Responses:** We appreciate the reviewers' careful evaluation. We have revised and condensed the following sentences, and merged them with the subsequent sentence for clarity: “Molecular epidemiologic investigations have shown that most gp85 genes in ALV-J layer strains formed an independent branch, and their homology with the ALV-J prototype strain HPRS103 was below 90.6%” (lines 58-60).

2. Although the authors have modified the discussion slightly, it is still too speculative and unnecessarily long. I recommend toning down the discussion of the structural implications of N123I substitution as it is based only on computative modeling, not actual structural analysis. I also advice to shorten the comparison of N123I with mutations increasing the receptor binding capacity of other viruses such as EboV and SARS-CoV-2.

**Responses**: We thank the reviewer for their careful view. Based on your suggestion, we have streamlined sentences in lines 268-274 and deleted and modified sentences in lines 275-278, and 280-287.

3. In the discussion, I concern two sentences about the occurrence of N123I in American and Chinese strains of ALV-J (lines 299 to 303). The discussion gives the impression that these are two independent cases; can this be supported by phylogenetic data?

**Responses**: We appreciate the reviewer's meticulous feedback. In this paragraph, we thoroughly examined the correlation between the N12I mutation and the epidemic outbreak, finding coherence between the two. Furthermore, our animal experiment trials underline that the N123I mutation increases the viral shedding rate. These results lead us to hypothesize that the N123I mutation may have a connection with the ALV-J outbreaks. In addition, based on your suggestion, to express the theme of this paragraph more clearly, we have modified the corresponding sentences in the revised manuscript (lines 297, 298, 300).

4. Although the authors declare proofreading by an English-speaking professional, I believe that the manuscript could have been smoother by extensive stylistic and argumentative editing.

**Responses:** We appreciate the reviewer's careful consideration. Our manuscript underwent English language editing by a scientifically versed, English-speaking professional at Editage (www.editage.cn).
